# Viral respiratory infections in a nursing home: a six-month prospective study

**DOI:** 10.1186/s12879-016-1962-8

**Published:** 2016-11-04

**Authors:** Tina Uršič, Nina Gorišek Miksić, Lara Lusa, Franc Strle, Miroslav Petrovec

**Affiliations:** 1 Institute of Microbiology and Immunology, Faculty of Medicine, University of Ljubljana, Zaloška 4, 1000 Ljubljana, Slovenia; 2 Department of Infectious Diseases, Maribor University Medical Center, Ljubljanska 5, 2000 Maribor, Slovenia; 3 Faculty of Medicine, Institute for Biostatistics and Medical Informatics, Vrazov trg 2, 1104 Ljubljana, Slovenia; 4 Department of Infectious Diseases, University Medical Center Ljubljana, Japljeva 2, 1525 Ljubljana, Slovenia

**Keywords:** Nursing home, Respiratory infections, Viral etiology, Nursing home residents, Nursing home caregivers, Visitors, Influenza A virus, Respiratory syncytial virus, Human metapneumovirus, Human rhinovirus/enterovirus

## Abstract

**Background:**

The knowledge on viral respiratory infections in nursing home (NH) residents and their caregivers is limited. The purpose of the present study was to assess and compare the incidence of acute respiratory infections (ARI) in nursing home (NH) residents and staff, to identify viruses involved in ARI and to correlate viral etiology with clinical manifestations of ARI.

**Methods:**

The prospective surveillance study was accomplished in a medium-sized NH in Slovenia (central Europe). Ninety NH residents and 42 NH staff were included. Nasopharyngeal swabs were collected from all participants at enrollment (December 5th, 2011) and at the end of the study (May 31st, 2012), and from each participant that developed ARI within this timeframe. Molecular detection of 15 respiratory viruses in nasopharyngeal swab samples was performed.

**Results:**

The weekly incidence rate of ARI in NH residents and NH staff correlated; however, it was higher in staff members than in residents (5.9 versus 3.8/1,000 person-days, *P* = 0.03), and was 2.5 (95 % CI: 1.36–4.72) times greater in residents without dementia than in residents with dementia. Staff members typically presented with upper respiratory tract involvement, whereas in residents lower respiratory tract infections predominated. Respiratory viruses were detected in 55/100 ARI episodes. In residents, influenza A virus, respiratory syncytial virus, and human metapneumovirus were detected most commonly, whereas in NH staff rhinovirus and influenza A virus prevailed. 38/100 ARI episodes (30/56 in residents, 8/44 in staff) belonged to one of three outbreaks (caused by human metapneumovirus, influenza A virus and respiratory syncytial virus, respectively). NH residents had higher chances for virus positivity within outbreak than HN staff (OR = 7.4, 95 % CI: 1.73–31.48, *P* < 0.01).

**Conclusions:**

ARI are common among NH residents and staff, and viruses were detected in a majority of the episodes of ARI. Many ARI episodes among NH residents were outbreak cases and could be considered preventable.

**Trial registration:**

The study was registered on the 1^th^ of December 2011 at ClinicalTrials (NCT01486160).

## Background

Respiratory infections represent about one-third of all infections in nursing home (NH) residents in Europe [1]. Pneumonia is the most common reason for hospital admittance among NH residents and a significant cause of mortality. It has been estimated that 40 % of lower respiratory tract community-acquired infections in persons >65 years old in the US are caused by viruses [2]. Each year about 54,000 deaths that occur in this population are due to infection with respiratory syncytial virus (RSV) and influenza virus (InfV) A and B [3, 4]. Infections caused by other viruses such as human rhinoviruses and human enteroviruses (hRV/EV), human metapneumovirus (hMPV), and human coronaviruses (hCoV) have also been reported, but data on the role of these viruses in the elderly are scant [5].

Infection with respiratory viruses may be asymptomatic or symptomatic; symptomatic infection may range from mild upper to severe lower respiratory tract infections, and may cause exacerbation of underlying diseases such as asthma and chronic obstructive pulmonary disease [6]. Viral respiratory infections can be sporadic or epidemic. RSV epidemics in adults usually alternate with epidemics of influenza and are clinically difficult to distinguish. Picornaviruses (hRV/EV) are predominantly associated with the common cold but are also the cause of lower respiratory tract infections, especially in small children and the elderly [7].] HMPV and hCoV are distributed worldwide, and in countries with temperate climates they usually cause winter epidemics, which often follow an RSV outbreak [8, 9]. Nevertheless, knowledge about infections with these viruses in NH residents is incomplete. In comparison to acute care hospitals, infection control practices in NH cannot be as strict; many vulnerable people with underlying chronic diseases share common spaces for daily activities and infections can spread easily. Due to daily visitors and NH staff, viral infections from the community can be introduced into the NH. In addition, scant clinical symptoms combined with a shortage of medical personnel and limited diagnostic facilities can lead to delayed recognition of infections and consequently delayed introduction of preventive measures.

The objectives of the study were to assess and compare the incidence of acute respiratory infections (ARI) in NH residents and NH staff, to identify viruses involved in ARI, and to correlate viral etiology with clinical manifestations of ARI in the population observed. We also planned to assess the correlation of the daily NH visitors on the occurrence of ARI in residents.

## Methods

### Study design and setting

We performed a prospective surveillance study from December 5th, 2011 to May 31st, 2012 in one of the sections of the 208-bed NH.

The study was conducted on the separated area of the NH, where ambulatory residents with dementia live together, on the floor, where residents live in single or two-bed rooms, and on the floor, where residents live in four-bed rooms. Whereas bedridden residents have meals served in their rooms, mobile residents have meals and other activities in a common dining room on each floor.

### Participants and data collection

Data were collected from 90/97 (93 %) residents to 42/53 (79.2 %) nursing care workers; all of them provided signed informed consent for participation. In participants with dementia a written consent from a legal representative was obtained.

Daily number of visitors (adults, school children, and preschool children, respectively) to NH residents’ rooms were recorded during the study period.

Nasopharyngeal swabs for virology studies were collected from all participants at the time of enrollment (December 5th, 2011) and at the end of the study (May 31st, 2012), as well as from each participant who developed ARI during the study.

Cases of ARI were defined according to McGeer criteria for infection surveillance in long-term care facilities [10] and were designated as upper respiratory tract infection (URTI) or lower respiratory tract infection (LRTI).

Detection of ARI was carried out daily by trained study nurses and the diagnosis was confirmed by a physician, who performed a physical examination in the case of illness. In each case of ARI, a nasopharyngeal swab was taken for diagnostic microbiology on the day when the illness was diagnosed.

For study purposes, outbreaks were defined as ≥2 cases of ARI among residents and/or employees within 5 days in the same NH unit, and with laboratory-confirmed infection with the same virus. The attack rate for an outbreak was calculated as the number of new cases of microbiologically confirmed ARI among residents during the outbreak per number of residents exposed. The number of residents exposed was estimated as the number of residents in contact with the infected person (sharing the same room, attending common activities, or using the dining room). The duration of an outbreak was defined as the time (days) elapsed from the onset of the illness in the first proven case to the onset of the illness in the last proven case.

### Laboratory methods

Automatic nucleic acid extraction was carried out using a total nucleic acid kit on a MagNa Pure Compact instrument (Roche Applied Science, Mannheim, Germany), following the manufacturer’s instruction.

Amplifications of parts of the specific genes of InfV A/B, adenovirus (AdV), parainfluenza viruses 1, 2, and 3 (PIV 1–3), hRV/EV, RSV, hMPV, hCoV (hCoV-229E, hCoV-NL63, hCoV-HKU1, and hCoV-OC43), and human bocavirus 1 (HBoV1) were performed using assays previously described [11–20].

PCR amplification of EV was conducted on a Light Cycler instrument 480 II (Roche, Applied Science, Mannheim, Germany) using an Enterovirus R-gene real-time PCR kit (ARGENE, Biomerieux, Verniolle, France) and performed following the manufacturer’s instructions.

### Statistical analyses

Categorical variables were summarized with frequencies and percentages, numerical variables with medians and interquartile ranges.

Incidence rates of ARI were calculated as events per 1,000 person-days, and their 95 % confidence intervals (CI) were based on the Poisson distribution. The comparison of ARI incidence rates between groups were based on univariable or multivarible Poisson regression models. Residents that died during the study were considered at risk until the time of death.

Test positivity to any respiratory virus (tested at a given time point) was compared between staff and NH residents using a univariable logistic regression model. To account for repeated measurements in individual subject, the analysis was adjusted for a subject variable using a random effect. The estimated odds ratio for any virus positivity for NH residents relative to staff members and its 95 % CI were reported.

The association between ARI incidence among NH residents and staff members, and between the number of visitors and number of ARI among NH residents, was evaluated with Kendall’s τ correlation.

## Results

The basic demographic data, clinical characteristics and influenza vaccination status of study participants are presented in Table 1. The median age of NH residents was 84, 72 % of residents were female. The majority (97 %) of residents had comorbidities.

**Table 1:** Basic characteristics of nursing home residents and staff members

Basic characteristics of study participants	NH residents	Staff members
Number of participants	90	42
Age (years), median (IQR)	84.0 (79.8–88.8)	38.2 (33.5–46.1)
Female, *n* (%)	65 (72.2)	41 (97.6)
Mobile capabilities:		
Bedridden, *n* (%)	27 (30)	0
Need assistance in daily activities, *n* (%)	32 (35.5)	0
Mobile, *n* (%)	31 (34.3)	42 (100)
Comorbidities^a^, *n* (%):	87 (96.7)	7 (16.7)
Cardiovascular disease, *n* (%)	76 (84.4)	3 (7.1)
Chronic pulmonary disease, *n* (%)	11 (12.2)	1 (2.4)
Cerebrovascular disease, *n* (%)	38 (42.2)	1 (2.4)
Diabetes mellitus, *n* (%)	25 (27.8)	3 (7.1)
Dementia, *n* (%)	46 (51.1)	0
Charlson comorbidity index (age-adjusted), median (IQR)	7 (5–9)	0
Smoking, *n* (%)	5 (5.6)	15 (35.7)
Influenza vaccination^b^ in 2011/2012 season, *n* (%)	58 (64.4)	9 (21.4)
Deaths^c^, *n* (%)	12 (13.3)	0

*IQR* interquartile range

^a^At least one comorbidity

^b^Vaccination included strains A/California/7/2009 H1N1, A/Perth/16/2009 H3N2, and B/Brisbane/60/2008

^c^Deaths during 6-month study period

At the beginning of the study, on December 5th, 2011, 42 staff members (33 healthy, nine with ARI) and 90 NH residents (only one with ARI) were tested for the presence of viruses in a nasopharyngeal swab. The predominant virus, detected in participants with ARI as well as in healthy participants, was hRV/EV (Table 2). Of 132 participants, 14 (13 residents and one member of staff) did not complete the 6-month study; the median follow-up in this subgroup was 79 (interquartile range: 27–123) days. In the nursing staff group, one of the nurses had a complicated pregnancy and was unable to work from the 127th day after the beginning of the study. During the study period, 12 NH residents died and one resident refused to attend the final testing.

**Table 2:** Respiratory viruses in nasopharyngeal swab detected in participants at the beginning and at the end of the study

	NH residents, healthy	NH residents with ARI	NH staff, healthy	NH staff with ARI
Beginning of study	89	1	33	9
Positive PCR (%)	9^d^ (10.1)	0	3^d^ (9.1)	2 (22.2)
Negative PCR (%)	80 (89.9)	1 (100)	30 (90.9)	7 (77.8)
End of study	73	4	39	2
Positive PCR (%)	3^e^ (4.1)	0	2^d^ (5.1)	1^d^ (50)
Negative PCR (%)	70 (95.9)	4 (100)	37 (94.9)	1 (50)
Total	162	5	72	11
Positive PCR (%)	**12 (7.4)**	0 (0)	**5 (6.9)**	3 (27.3)
Negative PCR (%)	150 (92.6)	5 (100)	67 (93.1)	8 (72.7)

^d^Rhinoviruses/enteroviruses, hRV/EV

^e^Parainfluenza virus type 3, coronavirus (could not be typed due to high Ct value) and hRV/EV, respectively, were detected

### Frequency of ARI

During the 6-month observation period, 100 episodes of ARI were observed: 56 among residents and 44 among staff. Of 42 residents with ARI, 29 (69.0 %) had a single episode of ARI, 12 (28.6 %) had two episodes, and one (2.4 %) had three episodes. The ARI incidence rate was 3.8/1,000 resident-days (95 % CI: 2.90–4.89). It was the highest in residents living in two-bed rooms (4.85 ARI/1,000 resident-days, 95 % CI: 3.28–7.30), followed by those living in single-bed rooms and in four-bed rooms (3.61, 95 % CI: 1.95–6.71, and 3.56, 95 % CI: 2.07–6.13, respectively), and the lowest in residents living in a separated area for demented mobile persons (2.53, 95 % CI: 1.32–3.87); the difference was not statistically significant (*P* = 0.36). Residents requiring assistance when walking and in daily activities had ARI more often (4.59 ARI/1,000 resident-days, 95 % CI: 3.1–6.79) than those who were either completely bedridden or mobile (3.28, 95 % CI: 2.04–5.29, and 3.29, 95 % CI: 1.95–5.56 ARI/1,000 resident-days, respectively); the difference was not statistically significant (*P* = 0.47).

Among 42 NH staff participants, 32 suffered from 44 episodes of ARI; mostly (21/32, 65.6 %) they had a single ARI episode, 10/32 (31.2 %) had two episodes, and one staff member (3.1 %) had three episodes of ARI. The ARI incidence rate among staff members was higher than in residents: 5.9 (95 % CI: 4.38–7.92) versus 3.8 (95 % CI: 2.90–4.89)/1,000 person-days (*P* = 0.03).

The weekly incidence rate of ARI in NH residents correlated with the corresponding incidence rate in NH staff (Kendall τ correlation coefficient 0.24, *P* = 0.14). In both groups the incidence was the highest in the 9th calendar week, whereas in the last third of the study period ARI cases were infrequent.

Using a multivariable Poisson model for the number of ARI occurrences, including age, influenza vaccination, mobility of residents, and chronic underlying diseases as explanatory variables, and controlling for the length of exposure, we estimated that the relative incidence-rate ratio of ARI for residents without dementia was 2.5 times greater than that for residents with dementia (95 % CI: 1.36–4.72); the other covariates were not statistically significantly associated with the number of ARI episodes.

### Etiology and clinical manifestations of ARI

**Fig. 1:**
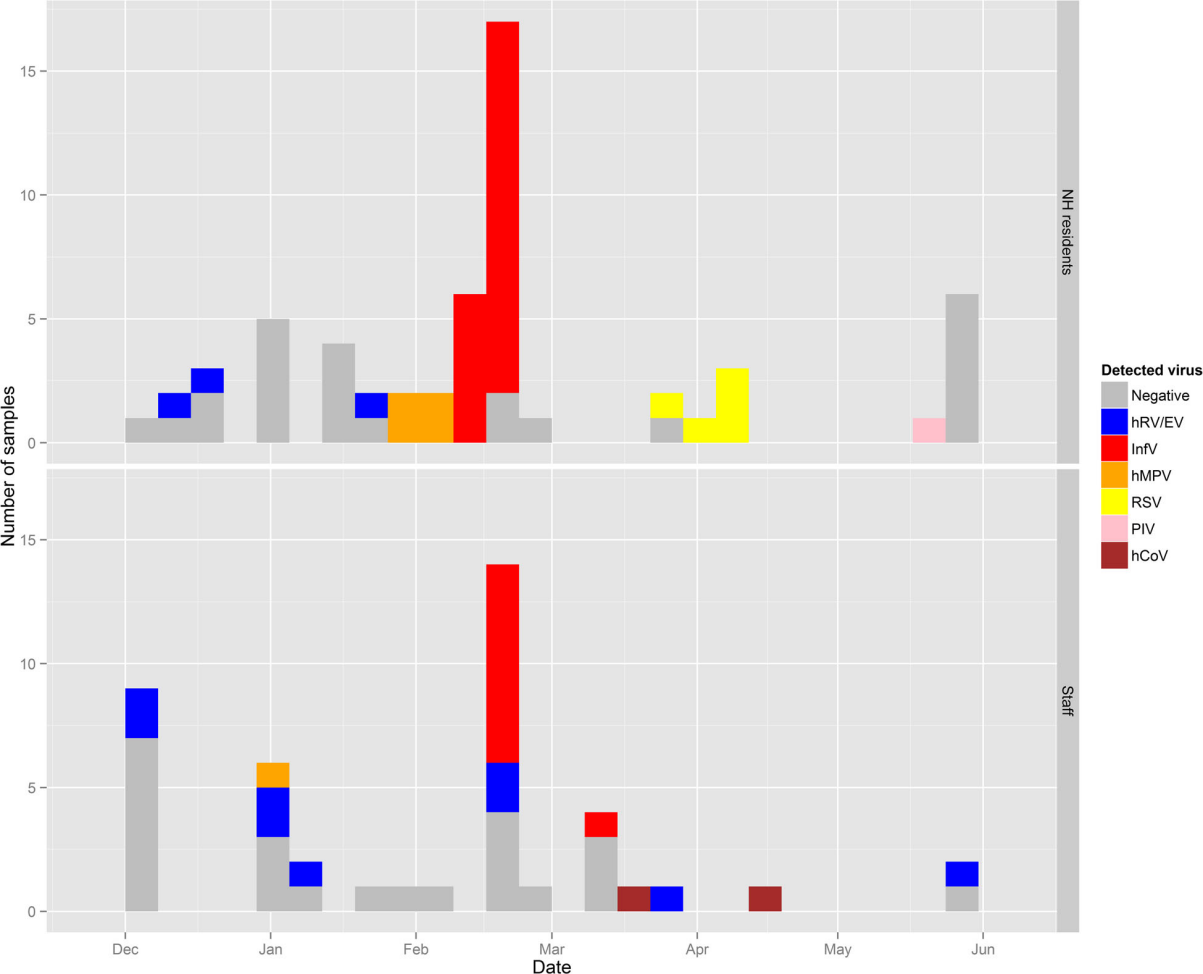
Demonstration of respiratory viruses in nursing home residents and staff with acute respiratory infection from December 5th, 2011 to May 31st, 2012

Respiratory viruses were detected in 55/100 (55 %) ARI episodes. Of these, 34/56 (60.7 %) cases occurred in residents, and 21/44 (47.7 %) cases occurred in staff (OR for virus positivity for NH residents relative to staff = 1.7, 95 % CI: 0.68–4.12, *P =* 0.27). Among residents, InfV A infection was detected most commonly (21/34, 61.8 %), followed by RSV (5/34, 14.7 %) and hMPV (4/34, 11.7 %), whereas in NH staff hRV/EV (9/42, 42.8 %) and InfV A (8/42, 38.1 %) were the most common (Table 3, Fig. 1). Only one virus was detected during an individual ARI episode in a particular person.

**Table 3:** Presence of respiratory viruses in nasopharyngeal swabs of nursing home residents and nursing home staff members with acute respiratory infections during the 6-month observational study

Viruses detected by PCR	Residents	URTI no. (%)	LRTI no. (%)	Staff	URTI no. (%)	LRTI no. (%)
ARI, *n*	56	15 (26.8)	41 (73.2)	44	43 (97.7)	1 (2.3)
PCR negative, *n* (%)	22 (39.3)	8 (53.3)	14 (34.1)	23 (52.3)	23 (53.5)	0
PCR positive, *n* (%)	34 (60.7)	7 (46.6)	27 (65.9)	21 (47.7)	20 (46.5)	1 (100)
RSV, *n* (%)	5 (8.9)	0	5 (12.2)	0	0	0
hRV/EV, *n* (%)	3 (5.0)	0	3 (7.3)	9 (18.2)	9 (20.9)	0
hMPV, *n* (%)	4 (7.1)	2 (13.3)	2 (4.9)	1 (2.3)	1 (2.3)	0
hCoV, *n* (%)	0	0	0	2 (4.6)	2 (4.7)	0
AdV, *n* (%)	0	0	0	0	0	0
hBoV, *n* (%)	0	0	0	0	0	0
PIV, *n* (%)	1 (1.8)	1 (6.7)	0	0	0	
InfV A, *n* (%)	21 (37.5)	4 (26.7)	17 (41.5)	9 (20.4)	8 (18.6)	1 (100)

*PCR* polymerase chain reaction, *URTI* upper respiratory tract infection, *LRTI* lower respiratory tract infection, *ARI* acute respiratory infection, *RSV* respiratory syncytial virus, *hRV/EV* human rhinovirus/enterovirus, *hMPV* human metapneumovirus, *hCoV* human coronavirus, *AdV* adenovirus, *hBoV* human bocavirus, *PIV* parainfluenza virus, *InfV A* influenza virus A

During the study period, three outbreaks were observed. The outbreak of hMPV infection started at the end of January and lasted for 9 days. Four residents living on the same floor developed ARI (two URTI, two LRTI; the attack rate among exposed residents was 26 %). In the middle of February an influenza A outbreak occurred; it lasted for 9 days. Infection with InfV A was established in 21 residents and eight staff members. Of them, nine presented with URTI (two residents and seven staff members) and 20 with LRTI (19 residents and one staff member); the observed attack rate was 23.3 % in residents, and 19.0 % in staff.

RSV outbreak was limited to residents. It started at the end of March, lasted for 13 days, and encompassed five residents. All presented with LRTI, one (20 %) of them died due to RSV infection. The attack rate among exposed residents was 11.6 %.

In this study, 38/100 ARI episodes belonged to one of the three outbreaks, whereas 62/100 ARI episodes were sporadic. Infections within outbreaks were more common in NH residents than in staff with ARI (30/56 versus 8/44, OR for virus positivity within an outbreak for NH residents relative to staff = 7.4, 95 % CI: 1.73–31.48, *P* < 0.01).

Of 56 ARI episodes in NH residents, 41 (73.5 %) fulfilled criteria for LRTI. In six (17.6 %) hospital admission was needed, and two residents died. The most common clinical signs were coughing (90.2 %) and a new finding on auscultation (95.1 %); fever was present in only 53 % episodes of LRTI. Viruses were detected in 27 (65.8 %) episodes of LRTI, most frequently InfV A (17/27, 63.0 %), followed by RSV (5/27, 18.5 %), hRV/EV (3/27, 11.1 %), and hMPV (2/27, 7.4 %) (Table 3).

Only 15/56 (26.8 %) episodes presented with URTI, including 11 (19.6 %) with common cold and four (7.1 %) with influenza-like illness. Residents suffered from runny nose (66.7 %), sore throat (66.7 %), and dry cough (60 %); fever was present in only 26.7 %. Viruses were detected in 7/15 (46.7 %) episodes of URTI, most commonly InfV A.

In contrast to residents, NH staff suffered almost exclusively from URTI (43/44 episodes of ARI); the only episode of LRTI in NH staff occurred during the influenza A outbreak. The most common clinical manifestation was common cold (74.4 %), followed by influenza-like illness (25.5 %). Viruses were detected in 46.5 % of the URTI episodes.

### Visitors and the occurrence of ARI

**Fig. 2:**
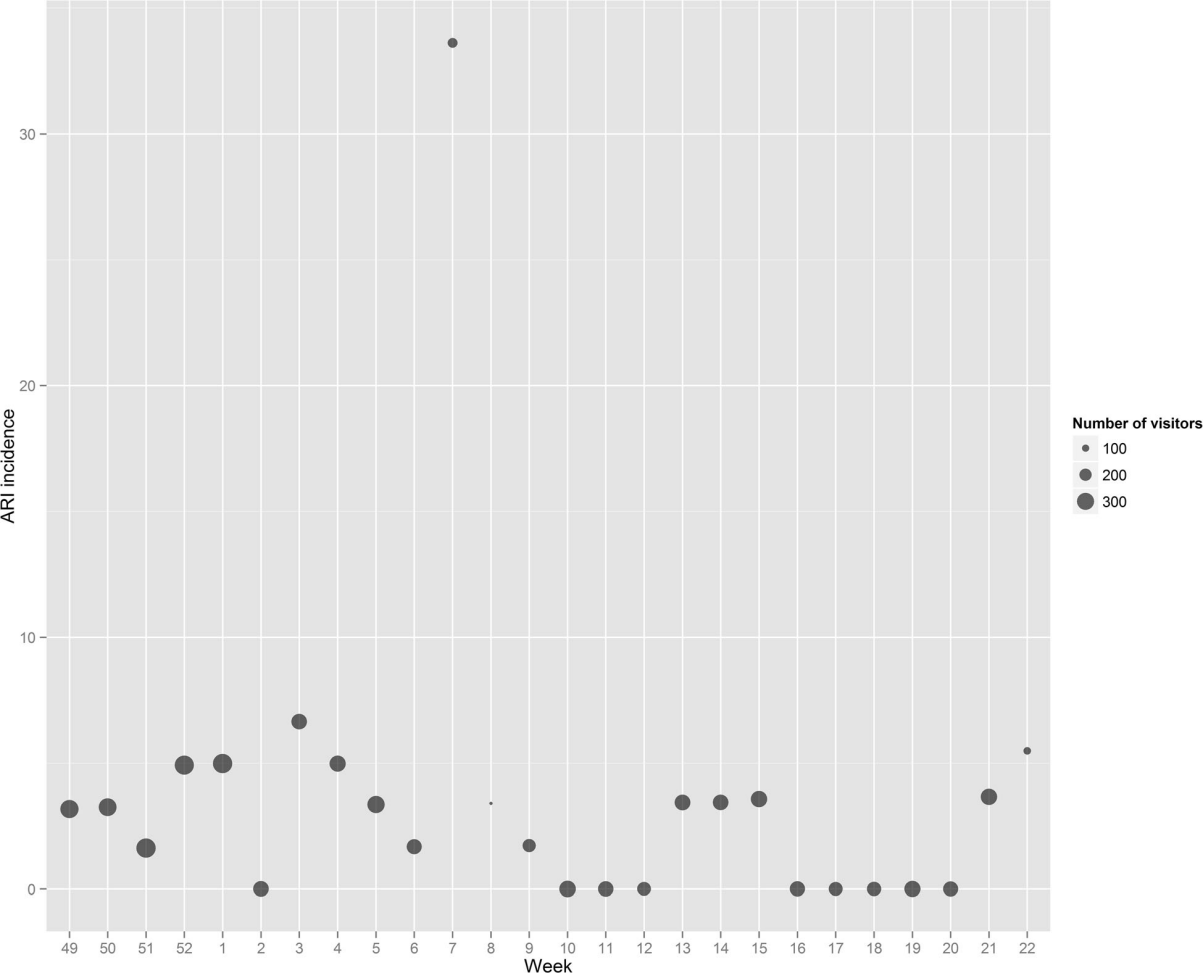
Weekly number of nursing home visitors and acute respiratory infections among residents

During the study period, 6,717 visitors to participants᾽ rooms were recorded: 96.3 % were adults, 2.3 % schoolchildren, and 1.3 % preschool children. From February 16th to 26th, the NH was closed to visitors because of influenza A outbreak. Data from this period were excluded from the following analyses. There were on average 268 visitors per week. The weekly number of NH visitors and weekly number of ARI among residents are shown in Fig. 2. Our data do not support the hypothesis of an association between the number of visitors and ARI (Kendall’s tau rank correlation = 0.12, *P* = 0.43); however, because visits were prohibited during the highest occurrence of ARI (during the influenza outbreak), the evaluation remained questionable.

## Discussion

Our study is one of a few prospective studies on ARI incidence in European NHs [1, 12, 13]. We observed a slightly lower incidence rate of ARIs among NH residents during winter than reported by Engelhart et al. [13] (3.8 (95 % CI: 2.90–4.89)/1,000 person-days vs. 4.4/1,000 person-days) but a higher incidence of LRTI as compared to reports from Norway [12] and Germany [13] (2.8 vs. 1.4 and 1.3/1,000 person-days, respectively). The ARI incidence rate in NH staff observed in this study (5.9, 95 % CI: 4.38–7.92/1,000 person-days) was comparable to the findings of Falsey [14] and was higher than in NH residents. This finding is in accordance with a study by Monto showing a decline in ARI incidence rate with increasing age of the observed population [15].

The most common manifestation of ARI among our NH residents was LRTI, representing 73.2 % of ARI episodes. Although a higher incidence rate of LRTI as compared to URTI in NH residents was rather expected, the LRTI vs. URTI ratio found in this study (2.7:1) was substantially higher than the ratio reported for NH residents in Germany (1.4:1) and in Wisconsin (1.7:1) [13, 16].

One of the interesting finding in our study was a significantly (2.5 times) lower ARI incidence rate among residents with dementia regardless of their mobility status, room type, number of visitors or comorbid conditions. In the demented residents the incidence of ARI could have been biased due to cognitive impairment, restraining them from reporting mild URTI symptoms, resulting in false lower ARI occurrence detection; however, ARI signs and symptoms were actively searched for every day by trained study nurses, and the ratio between URTI and LRTI in residents with dementia and the group without dementia was comparable (4/16 vs 11/25; *P* = 0.53). Potential other explanations for the lower ARI incidence rate could have been the result of a fewer number of visitors to demented in comparison to non-demented residents (not found in the present study) and/or less intensive personal contacts among family members and demented residents (not assessed in the present study). Nevertheless, in two European studies [17, 18] dementia was not found as a risk factor for respiratory tract infection among NH residents.

In this study, viruses were detected in 55 % of ARI episodes, in a higher proportion in residents than in staff (OR for virus positivity for NH residents relative to staff was 1.7, 95 % CI: 0.68–4.12; *P* = 0.27).

In residents, respiratory viruses were detected in 65.8 % of LRTI episodes. According to the published data, up to 40 % of LRTI in hospitalized elderly persons are linked to viral infections [2] and up to 23 % cases of radiological confirmed pneumonia in adults have a viral etiology [19–22]. We used a multiplexed real-time RT-PCR method, which is highly sensitive for detecting viruses in nasopharyngeal swabs [23, 24]; consequently, a higher percentage of viruses was detected among ARI episodes in our study as compared to studies not based on PCR methodology. Our findings in NH residents corroborate the findings of Liebermann et al., who reported a very high proportion of viruses involved in pneumonia (31.7 %) and in other LRTI (51.7 %) in adult patients [2]. However, the etiological role of viruses demonstrated in nasopharyngeal swabs in patients with ARI is associated with some ambiguity and is particularly problematic in patients with LRTI. Although the viruses may cause LRTI, they may also cause only URTI but not concurrently present LRTI (this may be due to bacterial superinfection, which viral URTI predisposes toward), or could simply be innocent bystanders. In this study, viruses were detected in nasopharyngeal swabs of 7.4 % healthy NH residents (mostly hRV/EV) and in 6.9 % of healthy NH staff (exclusively hRV/EV). These results are comparable with the 4 % occurrence of viruses in nasopharyngeal swabs obtained from healthy elderly persons living in the community [21] and with the findings of Liebermann et al. [2], who demonstrated viruses in nasopharyngeal swabs of 7.1 % of healthy adults.

In the present study only one virus was detected during an individual ARI episode in a particular person. This is in contrast to the findings in children with ARI in whom co-presence of more than one virus in the nasopharyngeal swab is a rather common finding [25].

Among viral respiratory pathogens, InfV and RSV are considered the most significant causes of excess morbidity and mortality among the elderly population. InfV A was the most common viral pathogen among NH residents in our study, followed by hMPV and RSV, which highlights the need for higher influenza vaccination coverage among NH residents and staff.

Of 56 ARI episodes that took place in NH residents during the 6-month period, 62 % were sporadic and 38 % occurred during three outbreaks, caused by InfV A (H3N2 subtype), RSV, and hMPV, respectively. The attack rates observed among residents ranged from 11.6 % for RSV infections to 26 % for hMPV infection. No hRV/EV outbreaks were observed during the study period; all hRV/EV infections were sporadic.

The relatively high rate of outbreak-associated ARI as compared to sporadic ARI corroborates results of Loeb et al. [26] Molecular diagnosis of a broader spectrum of pathogens as used in this study enabled us to identify two smaller outbreaks (hMPV, RSV), which would probably remained undetected without good surveillance, and etiologically undefined without the PCR diagnostic. Outbreaks of ARI were more common in residents than in NH staff, which emphasizes the importance of early recognition and good infection control practices in NHs.

One of the goals of this study was to assess the influence of visitor numbers on ARI incidence among NH residents. Because visits were prohibited during the influenza outbreak (i.e., during the highest occurrence of ARI), the appraisal remained indecisive. Another limitations of our study are that the investigation was performed only during the winter, when viral infections are more common, and that no bacterial causes were sought. With the extension of the analysis in the same nursing home over several seasons we would have been able to assess if the conclusions of present study had a more general appliance. Further limitations of the present study are the lack of viral sequencing (comparison of sequences would enable to reliably confirm the presumed links between the cases for each of the viruses), as well as small overall sample size and the low observed prevalence of most viruses, which drastically reduces the statistical power of any statistical comparison aimed at comparing specific viruses. For this reason we did not attempt to compare the prevalence of viruses among subgroups of participants.

## Conclusions

Respiratory infections are common among NH residents and NH staff, and viruses were detected in the majority of ARI episodes. Many ARI episodes among NH residents were outbreak cases and could be considered preventable especially because InfV A was the most commonly detected virus. Efforts in achieving higher vaccination rate among residents and especially among staff are needed.

## Abbreviations

ARI: Acute respiratory infection
CAP: Community-acquired pneumonia
CI: Confidence intervals
EV: Enteroviruses
HBoV1: Human bocavirus 1
hCoV: Human coronaviruses
hMPV: Human metapneumovirus
hRV/EV: Rhinovirus/enterovirus (picornaviruses)
InfVA: Influenza virus A
InfVB: Influenza virus B
LRTI: Lower respiratory tract infection
PCR: Polymerase chain reaction
RSV: Respiratory syncitial virus
URTI: Upper respiratory tract infection

## References

[1] Suetens C (2012). Healthcare-associated infections in European long-term care facilities: how big is the challenge?. Euro Surveill.

[2] Lieberman D, Shimoni A, Shemer-Avni Y, Keren-Naos A, Shtainberg R, Lieberman D (2010). Respiratory viruses in adults with community-acquired pneumonia. Chest.

[3] Falsey AR, Walsh EE, Hayden FG (2002). Rhinovirus and coronavirus infection-associated hospitalizations among older adults. J Infect Dis.

[4] Thompson WW, Shay DK, Weintraub E, Brammer L, Cox N, Anderson LJ, Fukuda K (2003). Mortality associated with influenza and respiratory syncytial virus in the United States. JAMA.

[5] Jartti L, Langen H, Soderlund-Venermo M, Vuorinen T, Ruuskanen O, Jartti T (2011). New respiratory viruses and the elderly. Open Respir Med J.

[6] Mahony JB (2008). Detection of respiratory viruses by molecular methods. Clin Microbiol Rev.

[7] Longtin J, Marchand-Austin A, Winter AL, Patel S, Eshaghi A, Jamieson F, Low DE, Gubbay JB (2010). Rhinovirus outbreaks in long-term care facilities, Ontario, Canada. Emerg Infect Dis.

[8] Boivin G, De Serres G, Hamelin ME, Cote S, Argouin M, Tremblay G, Maranda-Aubut R, Sauvageau C, Ouakki M, Boulianne N (2007). An outbreak of severe respiratory tract infection due to human metapneumovirus in a long-term care facility. Clin Infect Dis.

[9] Louie JK, Schnurr DP, Pan CY, Kiang D, Carter C, Tougaw S, Ventura J, Norman A, Belmusto V, Rosenberg J (2007). A summer outbreak of human metapneumovirus infection in a long-term-care facility. J Infect Dis.

[10] McGeer A, Campbell B, Emori TG, Hierholzer WJ, Jackson MM, Nicolle LE, Peppler C, Rivera A, Schollenberger DG, Simor AE (1991). Definitions of infection for surveillance in long-term care facilities. Am J infect cont.

[11] Daum LT, Canas LC, Arulanandam BP, Niemeyer D, Valdes JJ, Chambers JP (2007). Real-time RT-PCR assays for type and subtype detection of influenza A and B viruses. Influenza Other Respi Viruses.

[12] Eriksen HM, Koch AM, Elstrom P, Nilsen RM, Harthug S, Aavitsland P (2007). Healthcare-associated infection among residents of long-term care facilities: a cohort and nested case–control study. J Hosp Infect.

[13] Engelhart ST, Hanses-Derendorf L, Exner M, Kramer MH (2005). Prospective surveillance for healthcare-associated infections in German nursing home residents. J Hosp Infect.

[14] Falsey AR, McCann RM, Hall WJ, Tanner MA, Criddle MM, Formica MA, Irvine CS, Kolassa JE, Barker WH, Treanor JJ (1995). Acute respiratory tract infection in daycare centers for older persons. J Am Geriatr Soc.

[15] Monto AS (2002). Epidemiology of viral respiratory infections. Am J Med.

[16] Scheckler WE, Peterson PJ (1986). Infections and infection control among residents of eight rural Wisconsin nursing homes. Arch Intern Med.

[17] Heudorf U, Boehlcke K, Schade M (2012). Healthcare-associated infections in long-term care facilities (HALT) in Frankfurt am Main, Germany, January to March 2011. Euro Surveill.

[18] Eilers R, Veldman-Ariesen MJ, Haenen A, van Benthem BH (2012). Prevalence and determinants associated with healthcare-associated infections in long-term care facilities (HALT) in the Netherlands, May to June 2010. Euro Surveill.

[19] File TM (2003). Community-acquired pneumonia. Lancet.

[20] Johnstone J, Majumdar SR, Fox JD, Marrie TJ (2008). Viral infection in adults hospitalized with community-acquired pneumonia: prevalence, pathogens, and presentation. Chest.

[21] Graat JM, Schouten EG, Heijnen ML, Kok FJ, Pallast EG, de Greeff SC, Dorigo-Zetsma JW (2003). A prospective, community-based study on virologic assessment among elderly people with and without symptoms of acute respiratory infection. J Clin Epidemiol.

[22] Falsey AR, Walsh EE (2006). Viral pneumonia in older adults. Clin Infect Dis.

[23] She RC, Polage CR, Caram LB, Taggart EW, Hymas WC, Woods CW, Schmader K, Petti CA (2010). Performance of diagnostic tests to detect respiratory viruses in older adults. Diagn Microbiol Infect Dis.

[24] Templeton KE, Scheltinga SA, van den Eeden WC, Graffelman AW, van den Broek PJ, Claas EC (2005). Improved diagnosis of the etiology of community-acquired pneumonia with real-time polymerase chain reaction. Clin Infect Dis.

[25] Ursic T, Jevsnik M, Zigon N, Krivec U, Beden AB, Praprotnik M, Petrovec M (2012). Human bocavirus and other respiratory viral infections in a 2-year cohort of hospitalized children. J Med Virol.

[26] Loeb M, McGeer A, McArthur M, Peeling RW, Petric M, Simor AE (2000). Surveillance for outbreaks of respiratory tract infections in nursing homes. CMAJ.

